# Subclinical Primary Psychopathy, but Not Physical Formidability or Attractiveness, Predicts Conversational Dominance in a Zero-Acquaintance Situation

**DOI:** 10.1371/journal.pone.0113135

**Published:** 2014-11-26

**Authors:** Joseph H. Manson, Matthew M. Gervais, Daniel M. T. Fessler, Michelle A. Kline

**Affiliations:** 1 Department of Anthropology & Center for Behavior, Evolution and Culture, University of California Los Angeles, Los Angeles, California, United States of America; 2 Department of Psychological and Brain Sciences, and Sage Center for the Study of the Mind, University of California Santa Barbara, Santa Barbara, California, United States of America; 3 School of Human Evolution and Social Change, Arizona State University, Tempe, Arizona, United States of America; University of Goettingen, Germany

## Abstract

The determinants of conversational dominance are not well understood. We used videotaped triadic interactions among unacquainted same-sex American college students to test predictions drawn from the theoretical distinction between *dominance* and *prestige* as modes of human status competition. Specifically, we investigated the effects of physical formidability, facial attractiveness, social status, and self-reported subclinical psychopathy on *quantitative* (proportion of words produced), *participatory* (interruptions produced and sustained), and *sequential* (topic control) dominance. No measure of physical formidability or attractiveness was associated with any form of conversational dominance, suggesting that the characteristics of our study population or experimental frame may have moderated their role in dominance dynamics. Primary psychopathy was positively associated with quantitative dominance and (marginally) overall triad talkativeness, and negatively associated (in men) with affect word use, whereas secondary psychopathy was unrelated to conversational dominance. The two psychopathy factors had significant opposing effects on quantitative dominance in a multivariate model. These latter findings suggest that glibness in primary psychopathy may function to elicit exploitable information from others in a relationally mobile society.

## Introduction

When small groups of strangers are assembled for brief discussions, individual differences emerge along what Hall, Coats, and LeBeau [1] call “the vertical dimension…relating to power, dominance, status, hierarchy, and related concepts”. The generality in this formulation reflects a history of inconsistency in social scientists’ use of these terms [1], [2]. The term *dominance* is particularly polysemous. It may denote a characteristic of a dyadic relationship, specifically the identity of the individual who consistently wins one-on-one contests [3]. In this view, the individual-level determinants of dominance are an empirical rather than a definitional issue, and may include such surprising features as position in a genealogical structure [4], rather than any individual attribute. In contrast, personality psychologists (e.g. [5]) regard dominance as a stable individual trait, subsuming descriptors such as “assertive”, “forceful”, and “self-confident”.

Evolutionary approaches to human status asymmetries have distinguished between coercive and prosocial routes to resource acquisition (e.g. [6]). One version of this dichotomy is Henrich and Gil-White’s [7] distinction between *dominance* and *prestige* as processes whereby people acquire status (see also [8]). *Dominance* is a phylogenetically older system based on intimidation and coercion, whereas the *prestige* system is thought to be uniquely human, and based on freely-conferred deference [7]. In Henrich and Gil-White’s [7] model, dominant individuals use force to induce fear and avoidance in subordinates, whereas prestigious individuals possess socially valued skills and/or knowledge that attracts sycophants, who defer to them in order to gain proximity so as to facilitate social learning. Consistent with this formulation, dominance and prestige have been shown to be associated with different personality traits [9] and different testosterone profiles in men [10].

In the present study, we use the dominance/prestige contrast model [7] as a theoretical foundation for examining the determinants of conversational dominance in naturalistic interactions. *Conversational dominance* (e.g. [11], [12]) is an interaction-level phenomenon rather than a relationship-level or individual-level phenomenon. Itakura [12], [13] distinguishes among three forms of conversational dominance. *Quantitative* conversational dominance is demonstrated when a given individual speaks for more time, or utters more words, than her interlocutor(s). *Participatory* conversational dominance occurs when an individual interrupts his interlocutor(s) more often than he is interrupted. *Sequential* conversational dominance refers to a particular actor directing the flow of the conversation to a greater extent than her interlocutor(s). These interaction patterns, particularly quantitative dominance [14], [15] and participatory dominance [1], [16], are related to subjective ratings of dominance: people who talk more rate themselves as more dominant, and are also rated by others as more dominant. Some evidence [17] indicates that sequential dominance is also associated with perceived trait dominance. However, the relative roles of dominance and prestige processes in conversational dominance remain unexplored. More generally, naturally occurring conversation remains understudied from an evolutionary perspective. Evolutionary psychologists have relied largely on laboratory manipulations (e.g. work reviewed in [18]), whereas human ethologists have focused their attention on nonverbal social behavior (e.g. [19], [20]). Additionally, a handful of investigations have applied evolutionary perspectives to the analysis of textual material in mass media (e.g. [21]). However, little work has tested evolutionary hypotheses using data on the verbal aspects of naturally occurring social interactions. Indeed, to our knowledge, the only existing publication in which evolutionary approaches have been employed in the investigation of naturally occurring discourse is Malamuth and Thornhill’s [22] examination of the relationship between hostile masculinity, sexual aggression, and domineeringness in conversation. However, in that work, evolutionary theory merely provided a backdrop for considerations of the gendered nature of aggression rather than a unique source of predictions; likewise, “domineeringness” was evaluated using raters’ impressions of conversational behavior, rather than quantitative assessments thereof. Here, we use such precise metrics to test specific evolutionary hypotheses about the relationships between a variety of physical, social, and personality variables and conversational dominance, with the goal of illuminating its socio-relational, psychological, and functional bases.

We argue that observed conversational dominance may result from either zero-sum competition (*dominance*) or freely-conferred deference (*prestige*). On the one hand, when initially interacting with unfamiliar others, individuals should prioritize assessing the probability and likely outcome of an agonistic interaction. Even if the vast majority of interactions with strangers are peaceful and non-coercive, failure to make such assessments early on could leave an individual unable to adaptively deploy dominance and submission in the rare event of escalating tension; this risk warrants the uniform deployment of assessment upon first encounter. The same logic explains signaling and associated behaviors: if conflict is a possibility, consensus regarding relative rank benefits both dominant and subordinate individuals, since such concordance obviates the need for a direct contest. Conversational dominance may correspond to the unfolding of such low-cost assessment. Consonant with this position, Rosa and Mazur’s [23] classic study found that individuals who first broke eye contact with co-participants tended to produce fewer speech acts in a subsequent discussion than those who maintained eye contact. The authors interpret this result in terms of phylogenetically ancient dominance-submission signaling, arguing that initial eye contact establishes a dominance hierarchy that plays out in subsequent conversational behavior. Conversational dominance may also undermine prestige, to the extent that it reflects attempts to monopolize a conversation at the expense of other participants. Rather than conveying accessibility and attracting admirers, conversational dominance may rebuff learners seeking proximity and learning opportunities.

If conversational dominance in zero-acquaintance situations constitutes a form of dominance behavior *sensu* Henrich and Gil-White [7], then it should be positively associated with traits that are (1) reliably correlated with the ability and willingness to obtain resources by force or the threat of force, and (2) quickly and easily discernible. In men but not women, one such trait is physical formidability (strength, fighting ability, resource holding power/potential [24], [25]), which (1) is associated with greater anger-proneness and sense of entitlement, as would be expected if stronger men are better able than weaker men to coerce others into providing them with benefits [26], and (2) can be accurately judged even from still images of faces [24].

Furthermore, certain personality traits may underlie individuals’ pursuit of social dominance. Here we focus on one such trait, subclinical psychopathy. Psychopathy is a syndrome characterized by a constellation of traits and behaviors, including pathological lying, manipulativeness, grandiosity, shallow emotions, lack of empathy and remorse, impulsivity, irresponsibility, and frequent violation of social norms [27], [28]. Some research [28], [29] has supported a two-factor structure for psychopathy in which Factor 1 (F1, primary psychopathy) subsumes the interpersonal and affective aspects (e.g., manipulativeness and lack of empathy), while Factor 2 (F2, secondary psychopathy) subsumes the lifestyle and anti-social aspects (e.g., impulsivity and criminal conduct). These two factors are positively correlated at ∼0.40–0.50. Three-factor [30] and four-factor [31] models have also been proposed. Psychopathy is profitably conceptualized as dimensional rather than categorical [32], and variation in psychopathic personality traits can be detected in non-institutionalized populations using self-report instruments [29], [33]. Researchers have formulated and tested several evolutionary hypotheses for the maintenance of psychopathic traits in human populations, including frequency dependent selection [34], exploitation of social ecologies that promote high mobility and anonymous interactions [35], and strategic sensitivity to the expected relational value of social partners [36].

With respect to dominance motivation, psychopathy is characterized by a sense of grandiosity [27] and self-perceived relative rank [37], and recent work has implicated psychopathic traits (as part of the Dark Triad [38]) in the pursuit of dominance [39]. Research using Hawley’s [40] typology has shown that psychopathy is positively associated with both coercive (dominance-linked) and bistrategic (mixed coercive and prosocial) resource acquisition strategies [41].

With respect to capacity for conversational dominance, Cleckley [27] long ago linked psychopathy with a glib, charming self-presentation and hence with verbal intelligence. Distinct relationships have been found between the various psychopathy factors and verbal IQ scores. Among incarcerated children and adolescents, individuals who score higher on the interpersonal and impulsive/irresponsible psychopathy factors, but not on the affective factor, typically also score higher on tests of verbal and creative intelligence [42]. Based on the four-factor model, a study of male jail inmates found positive relationships between both verbal and performance IQ and the interpersonal and antisocial psychopathy factors, but negative relationships between IQ and the affective and lifestyle factors [43]. In contrast, another study found no relationship between verbal IQ and psychopathy in a psychiatric inpatient sample [44]. To date, no research has investigated whether any aspect of psychopathy is related to quantified verbal behavior in a natural or semi-natural social setting, despite its importance for understanding status asymmetries in human interaction.

Rather than reflecting competition for dominance *sensu* Henrich and Gil-White [7], conversational dominance may reflect inter-individual prestige differences. Interlocutors may grant more floor time to–or allow the conversation to be guided by–those that they perceive as having valuable knowledge. For example, research shows that when given a complex task for which one solution is demonstrably correct, individuals who more closely approach the correct solution when working alone (i.e., those with greater expertise) have more influence on solutions generated by group discussion, compared to individuals who have less expertise [45]. Furthermore, expertise is a stronger predictor of quantitative conversational dominance in groups that perform better at the task, compared to groups that perform worse [45]. Individuals in small task groups also differentiate between others’ substantive and non-substantive discussion contributions, in evaluating the usefulness of others’ participation [46]. Similarly, where such groups are not assigned a task, as in the present study, prestige-sensitive individuals might yield more floor time and topic control to co-participants who are perceived to possess more valuable knowledge in socially valued domains in general. This could reflect an evolved propensity to defer to prestigious individuals even in realms outside their area of expertise, as an across-the-board deference will generally earn learners greater access to successful models than will deference limited to particular domains [7].

Physical attractiveness, though not associated with possession of valuable knowledge, is an arena of prestige competition [47] and it positively affects interpersonal assessments [48] and (particularly in women) feelings of entitlement [26]. Though participants did not have direct access to our socioeconomic status (SES) proxy, research has shown [49] that the SES of college students can be reliably assessed by naïve observers based on 60-second slices of social interaction, suggesting that participants have some idea of their co-participants’ SES, a likely determinant of prestige. College class level may be a prestige marker within the local context of a gathering of undergraduates, because more advanced students presumably have greater knowledge and broader social connections than younger students, pertaining to important areas such as desirable courses, residence halls, extracurricular organizations, etc.

Contrary to the arguments underpinning our predictions, some research (e.g. [49]) supports the view that higher status (specifically, higher SES) individuals, being less dependent on others than low-SES individuals, will display more social disengagement, rather than more conversational dominance, during zero-acquaintance interactions. Our analyses afford the opportunity to test this general hypothesis as well as the hypotheses described above.

### Predictions to be tested

Our predictions fall into two broad categories: (1) those based on conversational dominance as an outcome of competition for dominance, and (2) those based on conversational dominance as a reflection of inter-individual prestige differences.

If conversational dominance in zero-acquaintance situations is an outcome of dominance competition, then men who are (1) taller, and who are judged by physical appearance to be (2) stronger and (3) more likely to win fights against male opponents, will be more likely to dominate conversations. In both sexes, primary (F1) psychopathy, but not secondary (F2) psychopathy, will be positively associated with conversational dominance. We base this prediction on a meta-analysis [50] of psychopathy as a personality configuration. Both psychopathy factors were negatively correlated with Agreeableness and Conscientiousness, but only F2 was also negatively correlated with Extraversion and positively correlated with Neuroticism, whereas F1 was uncorrelated with both dimensions. Thus, in a zero-acquaintance situation, after controlling for the other psychopathy factor, (1) primary psychopathy, which encompasses manipulativeness and superficial charm, will be positively associated with measures of conversational dominance, whereas (2) secondary psychopathy, being associated with low Extraversion and high Neuroticism, will be negatively associated with measures of conversational dominance. People higher in F1 psychopathy may also be adept at eliciting talk from others, as a way of gathering useful information about them. We therefore predict a positive relationship, across conversation groups, between the F1 level of the participant with the highest F1 level and the rate of aggregate (group-level) word production. Finally, we examine whether F1 psychopathy is negatively associated with the use of affect words. Shallow emotional experience is a prominent feature of primary psychopathy [28], and several researchers (e.g. [51], [52]) have found associations between personality and word use.

If conversational dominance in zero-acquaintance situations is a reflection of inter-individual prestige differences (i.e., if it constitutes a form of freely-conferred deference), then it should reflect differences in locally relevant expertise or observable cues to skill, success, and deference from others [7]. We therefore examine the relationship between conversational dominance and five proxy measures of prestige: (1) physical attractiveness, (2) socioeconomic status (SES), as measured by median income in childhood ZIP code (a common practice in public health research, e.g. [53]), (3) social status as judged by third parties from clothing, hairstyle, personal adornment, and/or skin color (i.e., from images lacking identifiable faces), (4) perceived prestige of self-reported academic major, and (5) college class level.

## Materials and Methods

### Conversation participants

Conversation participants included 88 college students recruited from the participant pool of a large introductory course who received course credit for participation, 15 people recruited via flyers posted on campus, and two people whose recruitment origin could not be determined. Only native speakers of English were recruited. The publicized study title was “Small Talk Among Strangers”. All participants, regardless of recruitment source, received $10 compensation. Participants were scheduled in groups of same-sexed individuals such that either (1) all the members of a group, (2) none of them, or (3) only one of them had been recruited from the introductory course. Of the 35 conversational triads, 20 were all-female and 15 were all-male. The participants’ ethnic mix accurately reflected that of the college’s undergraduate population (see [36]). The median participant age was 19 years.

### Conversation task

Upon arrival at the laboratory, conversation participants were directed to cubicles where they were kept visually isolated from one another until they simultaneously entered the conversation room. In this room, they were invited to sit in chairs grouped equidistantly around a low table. Each participant’s chair location was indicated by a pre-assigned, randomly determined letter code. After determining that the conversation participants were strangers to each other, the experimenter recited a prepared script asking the participants to converse for 10 minutes on any topic(s) they wished. Conversation participants were informed that their conversation would be videotaped, but they were told nothing specific about the post-conversation portion of the procedure (see below), only that they would be asked some questions pertaining to social behavior. The experimenter then turned on the video camera, left the room, and closed the door.

### Other data collected from conversation participants

At the end of the 10 minute period, an experimenter re-entered the conversation room and asked the participants to return to their cubicles and not to interact for the remainder of the experiment. Seated at laptops, participants were instructed to begin the questionnaire phase of the experiment. First, each participant played a one-shot prisoner’s dilemma game, with $6–12 at stake, toward each of her co-participants (for these results see [36]). Second, participants were asked to complete the Levenson Self-Report Psychopathy Scale (LSRP), a 26-item instrument [29]. The items elicit agreement or disagreement on a 4-point Likert scale with statements such as “I let others worry about higher values; my main concern is with the bottom line” and “I am often bored”. The LSRP has been found to reproduce the hypothesized two-factor structure of psychopathy (see above); 16 items load on F1 and 10 items load on F2. The LSRP predicts performance on a psychopathy-linked response modulation task in undergraduate populations [33]. After completing the LSRP, participants were asked to indicate their age, sex, ethnicity, and childhood ZIP code or hometown. The entire procedure was programmed and conducted with the software z-Tree version 2.1 [54]. Finally, participants were photographed individually (clothed full-body photographs), after being instructed to assume a relaxed, neutral facial expression, using a Sony DCS-W200 12 megapixel (4000×3000) camera. Photographs were taken without flash, in day white florescent light (N 4600–5400 K), at a distance of 2.15 m using a focal length of 7.6–8.0 mm. We took two photographs of each participant, and used the one that we judged to be of higher quality (sharper focus, more neutral expression, etc.).

### Raters

Four sets of participants judged attributes of the conversation participants. *Attractiveness raters* judged facial attractiveness, *formidability raters* judged strength or fighting ability, *prestige raters* judged the prestige of academic majors claimed by conversation participants, and *social status raters* judged pairs of conversation co-participants with respect to relative social status. Characteristics of the rater samples are described in text S1.

### Rater stimuli and procedures

Complete details regarding the stimuli presented to raters, and the procedures by which raters judged the stimuli, are presented in text S1. Briefly, stimuli presented to the attractiveness raters were faces cropped from the full-body photographs taken of the conversation participants. They were rated on a 6-point Likert scale. The formidability raters used a 7-point Likert scale to rate the physical strength or fighting ability of full-body images de-identified by blurring the face in order to protect participants’ privacy during online presentation. Prestige raters rank-ordered the prestige of the 39 academic majors that were claimed by at least one conversation participant. Social status raters viewed pairs (comprised of two participants drawn from the same conversation triad) of the same de-identified full-body images that were presented to the formidability raters, and were asked to compare the two people in terms of social status on a 7-point Likert scale. Finally, a research assistant used the full-body images, and a metric marker on the wall in front of which conversation participants had been photographed, to estimate each participant’s height.

### Ethics Statement

All procedures were approved by the UCLA Institutional Review Board (Approvals #G07-10-097-01 to -04; #G10-01-004-01; #10-000371; and #10-001179). With the exceptions of the formidability raters and the prestige raters, written informed consent was obtained from all participants in accordance with the terms of these approvals. Because the formidability raters were internet respondents, and the prestige raters provided no individually identifying information, and because the procedures were judged to entail minimal risk, the UCLA IRB exempted those studies from the requirement of written informed consent. Formidability raters read a consent statement and clicked a link to begin the survey, thereby indicating their consent.

### Data archiving

Data for the study described in this paper are archived at www.escholarship.org/uc/item/2bx584t4.

### Conversation transcription and coding

Because of the large time and training investment required to transcribe conversational material at the desired level of detail (see below), a portion of each 10-minute conversation was pre-selected for transcription and further analysis. This portion always included the first 60 seconds of the conversation. Two additional transcription periods of at least one minute duration were randomly chosen from minutes 1–5 and 5–10, respectively, of each conversation. The amount of time transcribed per 10-minute conversation ranged from 3.02–5.57 minutes (*M* = 4.08, *SD* = 0.68 minutes). See [36] for more details.

Author JHM transcribed this material using the Conversation Analysis transcription system originally developed by Jefferson [55]. This transcription system includes finely detailed recording of the onsets and offsets of speech overlaps. Approximately one hour of work is required to transcribe one minute of talk, and the technique requires extensive specialized training [56], [57]. While ignorant of the participants’ LSRP responses and all other post-conversation responses, JHM coded the transcribed portions of the conversations for the occurrence of a number of features, including interruptions, which were defined as overlaps beginning at points that are not transition-relevant. Most overlaps begin at points when the second speaker may reasonably project that the first speaker has finished, or is about to finish, a turn [55]. Cues to projectable turn completion (and hence transition-relevance) may be grammatical or prosodic, but fundamentally a turn is projectable as complete when a listener can infer that its social action (asking, telling, assessing, etc.) is finished or nearly finished.

JHM also coded the transcribed conversation segments for the occurrence of sequence starts. These were defined as initiations of any of the following sequence types (see [58]): Greeting-greeting; self-introduction-self-introduction; self-description-self-description; question-answer; assessment-assessment; telling-acknowledgment; telling-telling; complaint-response; and self-deprecation-response. A research assistant was trained in the coding scheme and was assigned nine randomly chosen conversations for which to independently code sequence starts to check rater reliability.

Finally, to measure the frequency of affect words produced by each participant, we constrained the transcriptions to yield only English words spelled as indicated in the Linguistic Inquiry and Word Count (LIWC) 2007 program dictionary [59]. The LIWC calculates, for a sample of speech or text, the proportion of words in a text that fall into each of 67 categories, not all of which are mutually exclusive.

### Data analysis

#### Dependent measures

The three conversational dominance measures were calculated as (1) an individual’s proportion of the triad’s words uttered, hereafter *proportion of words uttered* (quantitative dominance), (2) proportion of the triad’s sequence starts performed, hereafter *proportion of sequence starts* (sequential dominance), and (3) interruptions performed per transcribed minute, hereafter *interruption rate* (participatory dominance). To control for baseline talkativeness with respect to sequence starts and interruptions, we also constructed two conversational dominance measures that controlled for word production: (A) sequence starts per word produced (multiplied by 100 for ease of viewing), and (B) interruptions performed per word produced (also multiplied by 100). Finally, because interruption rate (skewness = 1.87) and interruptions per word (skewness = 1.64) were strongly right-skewed, they were Box-Cox transformed before regression analysis.

#### Independent variables: dominance competition predictions

Conversation participants’ strength and fighting ability scores were calculated as the mean rating, across formidability rates, of each attribute. Strength, fighting ability and estimated height were standardized (converted to Z-scores) separately within each gender. Because participants were wearing their own clothing rather than assigned, invariant clothing (as in [24]), we examined whether upper body clothing type (long-sleeved, short-sleeve or tank-top) affected strength or fighting ability ratings and/or their relationships with conversational dominance measures. Each conversation participant’s total LSRP score, Factor 1 LSRP score, and Factor 2 LSRP score were first calculated as the mean response across all relevant LSRP items. LSRP scores were standardized across the entire sample.

#### Independent variables: prestige difference predictions

Conversation participants’ facial attractiveness scores were calculated as the mean rating across attrativeness raters. These scores were standardized separately within each gender. We used the median ranking of each conversation participant’s academic major as her major prestige score. For every participant who provided a childhood ZIP code (*N* = 101), we consulted the U.S. Census Bureau’s 2000 database (U.S. Census Bureau) to find that ZIP code’s median annual household income. We did not standardize this variable. Perceived status differences (from the de-identified full-body images) were standardized.

#### Reliability

We assessed some inter-rater reliabilities using a model of intra-class correlation for random effects based on repeated measures ANOVA [60]. Specifically, employing the icc23 command in Stata 12.0, we used the two-way mixed model (ICC [3, k]) in which stimuli are random, but raters are fixed. The inter-rater reliability of the academic major prestige rankings was assessed using Cronbach’s alpha.

#### Statistical tests of predictions

To assess relationships among independent variables, and to assess relationships among the three conversational dominance measures, we calculated Pearson correlation coefficients when only interval-scale variables were involved, and Spearman’s (nonparametric) rank correlation coefficients for all tests including the academic major prestige rank variable. As a first step toward assessing relationships between independent variables and conversational dominance measures, we used univariate linear regressions. To examine effects of facial attractiveness and physical formidability on conversational dominance, we ran four sets of analyses. First, we used, as data points, individual participants’ scores on all independent variables (attractiveness, height, strength, fighting ability, prestige measures) and uncorrected dependent variables (proportion of words uttered, proportion of sequence starts, and interruption rate). Second, reasoning that participants’ conversational behavior might be affected by their assessment of co-participants’ facial attractiveness and/or physical formidability relative to their own, we used dyads as data points, and examined relationships among the difference scores in one independent variable (e.g., the difference between *A*’s and *B*’s attractiveness scores) and one uncorrected dependent variable (e.g., the difference between proportion of sequence starts by *A* and by *B*). Third, we examined relationships between individual attractiveness and formidability scores (independent variables) and the two conversational dominance measures that were corrected for overall word production: (1) interruptions performed per word uttered, and (2) sequence starts begun per word uttered. Finally, we ran analyses using dyadic difference scores of the word-count-corrected measures.

In all regression analyses in which the scores of individuals or dyads within a conversational triad were not independent, we calculated robust standard errors of the regression coefficients, clustering by conversational triad. Variables with non-independent scores include all those involving proportion of words uttered, proportion of sequence starts, and all dyadic difference scores. All tests were two-tailed. We used Bonferroni-corrected α levels whenever we carried out multiple tests of the same hypothesis.

Finally, we ran multivariate models, using independent variables that successfully predicted our dependent variables, and used Akaike’s Information Criterion (AIC: [61]) to assess the relative quality of these models. In a comparison between two models predicting the same dependent variable, a lower AIC score indicates a better approximation to underlying causal processes without over-fitting to the data.

## Results

### Reliability

Inter-rater reliability of the coding of interruptions is reported in [36]. Records of the number of words produced by each participant (*N* = 18) were strongly correlated between the two coders (*r* = 0.99). Records of the number of sequence starts produced by each participant (*N* = 27) were acceptably correlated between the two coders (*r* = 0.75). Intra-class correlation analyses yielded a reliability (ICC [3, k]) across raters of 0.89 for mean attractiveness ratings of conversational participants, 0.94 for mean male strength ratings, 0.79 for mean male fighting ability ratings, 0.84 for mean female strength ratings, 0.86 for mean female fighting ability ratings, and 0.70 for relative status within conversation dyads. Of the 42 conversation participants whose height was estimated by both author MG and the research assistant, height estimates were within 1 cm for 40 (95%) of them. Cronbach’s alpha for the prestige raters’ rankings of academic majors was 0.90.

### Associations among independent variables

Interval-scale independent variables included estimated height, mean rated strength, mean rated fighting ability, mean rated attractiveness, total LSRP score, F1 LSRP score, F2 LSRP score, and median income of childhood ZIP code. Perceived strength and perceived fighting ability were strongly positively correlated in both women (*N* = 60, *r* = 0.74, *P*<0.001) and men (*N* = 45, *r* = 0.77, *P*<0.001). F1 and F2 LSRP were significantly correlated with each other (*N* = 105, *r* = 0.38, *P*<0.001) and, unsurprisingly, with total LSRP (F1: *r* = 0.93; F2: *r* = 0.70; both *P*<0.001). Considering both sexes of conversation participants, we found no other significant correlations. Among females only, taller individuals were judged to be stronger (*r* = 0.25, *P* = 0.05) and to have greater fighting ability (*r* = 0.27, *P*<0.05). Among males only, individuals with greater fighting ability had higher F2 LSRP scores (*r* = 0.33, *P*<0.05), whereas taller men had lower F1 LSRP scores (*r* = −0.33, *P*<0.05). We used a non-parametric correlation (Spearman’s rho) to test for associations between median prestige rank of academic major and the other independent variables. None of these correlations was significant at the individual level. At the level of within-dyad differences, the individual with the more prestigious major was actually viewed as having lower status than the individual with the less prestigious major (*N* = 91, ρ = 0.222, *P*<0.05).

### Correlations among conversational dominance measures

Among individuals, proportion of words uttered was strongly correlated with proportion of sequence starts (*N* = 105, *r* = 0.57, one-tailed *P*<0.001) and significantly though less strongly with interruptions performed per minute (*r* = 0.17, *P* = 0.04). Proportion of sequence starts was uncorrelated with interruption rate (*r* = 0.06, *P* = 0.27).

### Physical formidability and conversational dominance

Generally, there were no reliable effects of physical formidability on conversational dominance. Table 1 shows regression coefficients and 95% confidence intervals for all 15 tests of this general relationship in male individuals, while Table 2 shows the same analyses for male dyadic difference scores. No relationship was significant (i.e., all confidence intervals included zero), and 5 of the relationships were negative. Though we had made no predictions about female physical formidability and conversational dominance, we carried out the same regressions for females, again finding no results that approached significance. Uncontrolled variation in upper body clothing type (cf. [24]) did not appear to be responsible for these null results (see text S2).

**Table 1:** Linear regressions of conversational dominance variables on standardized male formidability variables.

Independent variable	Dependent variable	β ± robust SE	95% CI
Height	Words^a^	−0.02±0.02	−0.07–0.02
Height	Interruptions^b^	0.00±0.13	−0.25–0.26
Height	Sequence starts^c^	−0.02±0.02	−0.06–0.02
Height	Interruptions/words^d^	0.06±0.21	−0.29–0.63
Height	Sequence starts/words^e^	−0.13±0.16	−0.27–0.38
Perceived strength	Words^a^	0.02±0.02	−0.03–0.07
Perceived strength	Interruptions^b^	0.17±0.12	−0.08–0.41
Perceived strength	Sequence starts^c^	0.04±0.03	−0.02–0.10
Perceived strength	Interruptions/words^d^	0.07±0.16	−0.25–0.40
Perceived strength	Sequence starts/words^e^	0.12±0.12	−0.15–0.38
Perceived fighting ability	Words^a^	−0.02±0.02	−0.07–0.03
Perceived fighting ability	Interruptions^b^	0.02±0.13	−0.24–0.27
Perceived fighting ability	Sequence starts^c^	0.02±0.04	−0.05–0.10
Perceived fighting ability	Interruptions/words^d^	−0.05±0.20	−0.48–0.38
Perceived fighting ability	Sequence starts/words^e^	0.20±0.13	−0.07–0.47

*N* = 45 individuals.

a proportion of triad’s words uttered.

b interruptions per transcribed minute, Box-Cox transformed.

c proportion of triad’s sequence starts.

d interruptions performed per word uttered×100, Box-Cox transformed.

e sequence starts per word uttered×100.

**Table 2:** Linear regressions of conversational dominance variables on standardized male formidability variables.

Independent variable	Dependent variable	β ± robust SE	95% CI
Height	Words^a^	−0.03±0.03	−0.10–0.04
Height	Interruptions^b^	0.23±0.36	−0.54–1.00
Height	Sequence starts^c^	−0.02±0.03	−0.08–0.03
Height	Interruptions/words^d^	0.08±0.19	−0.32–0.48
Height	Sequence starts/words^e^	−0.13±0.18	−0.53–0.26
Perceived strength	Words^a^	0.03±0.04	−0.05–0.11
Perceived strength	Interruptions^b^	0.07±0.29	−0.56–0.71
Perceived strength	Sequence starts^c^	0.07±0.04	−0.01–0.15
Perceived strength	Interruptions/words^d^	−0.17±0.24	−0.68–0.35
Perceived strength	Sequence starts/words^e^	0.09±0.23	−0.39–0.58
Perceived fighting ability	Words^a^	−0.03±0.05	−0.13–0.07
Perceived fighting ability	Interruptions^b^	−0.25±0.30	−0.89–0.39
Perceived fighting ability	Sequence starts^c^	0.04±0.05	−0.08–0.16
Perceived fighting ability	Interruptions/words^d^	−0.28± 0.21	−0.73–0.17
Perceived fighting ability	Sequence starts/words^e^	0.28±0.21	−0.18–0.74

*N* = 45 dyadic difference scores.

a proportion of triad’s words uttered.

b interruptions per transcribed minute.

c proportion of triad’s sequence starts.

d interruptions performed per word uttered×100.

e sequence starts per word uttered×100.

### Prestige measures and conversational dominance

There were no reliable effects of facial attractiveness on conversational dominance. Table 3 shows regression coefficients and 95% confidence intervals for all 10 tests of this general relationship in women, while Table 4 shows the same analyses for men. No relationship was significant (i.e., all confidence intervals included zero), and 10 of the relationships (7 in women, 3 in men) were negative.

**Table 3:** Linear regressions of conversational dominance variables on standardized female facial attractiveness.

	Individuals		Dyadic difference scores	
	β ± robust SE	95% CI	β ± robust SE	95% CI
Independent variable				
Words^a^	0.00±0.01	−0.03–0.03	0.02±0.03	−0.04–0.07
Interruptions^b^	−0.06±0.11	−0.28–0.16	−0.32±0.29	−0.94–0.29
Sequence starts^c^	0.00±0.02	−0.04–0.04	−0.01±.03	−0.08–0.07
Interruptions/words^d^	−0.12±0.13	−0.38–0.15	−0.18±0.14	−0.47–0.12
Sequence starts/words^e^	−0.07±0.10	−0.29–0.15	v0.18±0.28	−0.77–0.41

*N* = 60 for both individuals and dyadic difference scores.

a proportion of triad’s words uttered.

b interruptions per transcribed minute, Box-Cox transformed.

c proportion of triad’s sequence starts.

d interruptions performed per word uttered×100, Box-Cox transformed.

e sequence starts per word uttered×100.

**Table 4:** Linear regressions of conversational dominance variables on standardized male facial attractiveness.

	Individuals		Dyadic difference scores	
	β ± robust SE	95% CI	β ± robust SE	95% CI
Independent variable				
Words^a^	0.00±0.02	−0.05–0.04	0.02±0.03	−0.04–0.07
Interruptions^b^	0.08±0.13	−0.18–0.33	0.09±0.42	−0.81–0.98
Sequence starts^c^	−0.01±0.02	−0.07–0.04	−0.03±0.05	−0.12–0.07
Interruptions/words^d^	0.11±0.16	−0.22–0.44	0.39±0.19	−0.02–0.81
Sequence starts/words^e^	–0.10±0.16	−0.45–0.24	0.10±0.27	−0.48–0.68

*N* = 45 for both individuals and dyadic difference scores.

a proportion of triad’s words uttered.

b interruptions per transcribed minute, Box-Cox transformed.

c proportion of triad’s sequence starts.

d interruptions performed per word uttered×100, Box-Cox transformed.

e sequence starts per word uttered×100.

There were no significant relationships between median household income and either proportion of words uttered or interruption rate. Participants from wealthier ZIP codes performed fewer sequence starts (*N* = 101, β = −0.0015±0.0005, *P* = 0.008, Bonferroni-adjusted α for three tests = 0.017). To aid interpretation of effect sizes, ZIP code median income was not standardized. Thus, this result means that for every additional $1000 in median annual household income, a conversation participant’s expected proportion of sequence starts decreased by 0.0015. Sequence starts per word uttered was also negatively associated with ZIP code median income (β = −0.010±0.004, *P* = 0.014). Results of dyadic difference scores were consistent with these findings: the participant from a higher income ZIP code tended to produce a smaller proportion of sequence starts compared to his or her co-participant from the lower-income ZIP code (β = −0.004±0.001, *P* = 0.006), even when corrected for number of words uttered (β = −0.0171±0.008, *P* = 0.032), though the latter result is not significant after Bonferroni adjustment. Dyadic difference score analysis also revealed a trend for the participant from the higher income ZIP code to produce a smaller proportion of words compared to his or her co-participant from the lower-income ZIP code (β = −0.0023±0.001, *P* = 0.028). This trend was not significant after Bonferroni adjustment.

Perceived dyadic social status difference, as judged from the pairs of images of co-participants, was unrelated to dyadic difference in (1) proportion of words uttered (*N* = 105, β = 0.046±0.032, *P* = 0.16), (2) interruption rate (β = 0.472±0.288, *P* = 0.11), (3) proportion of sequence starts (β = −0.026±0.04, *P* = 0.519), (4) interruptions per word uttered (β = −0.186±0.150, P = 0.224) and (5) sequence starts per word uttered (β = 0.094±0.236, *P* = 0.692).

There were no significant effects of median academic major prestige ranking on the five conversational dominance measures (*N* = 99) at either the individual or dyadic difference level (maximum value of ρ: 0.042). Of the 105 conversation participants, 98 (93.3%) announced their class level at some point during the conversation. In 23 of the 35 triads, one participant was at a more advanced class level than either of his or her co-participants. These more advanced students produced a marginally higher proportion of their triad’s words than did the less advanced students of these triads (*N*_advanced_ = 23, *N*_non-advanced_ = 46, *M*_advanced_ = 0.377, *M*_non-advanced_ = 0.312, *t* = 1.92, *P* = 0.058, Cohen’s *d* = 0.48). Relative class level was not associated with proportion of sequence starts or interruption rate, whether or not these measures were corrected for number of words uttered.

### Psychopathy factors and conversational dominance

**Figure 1:**
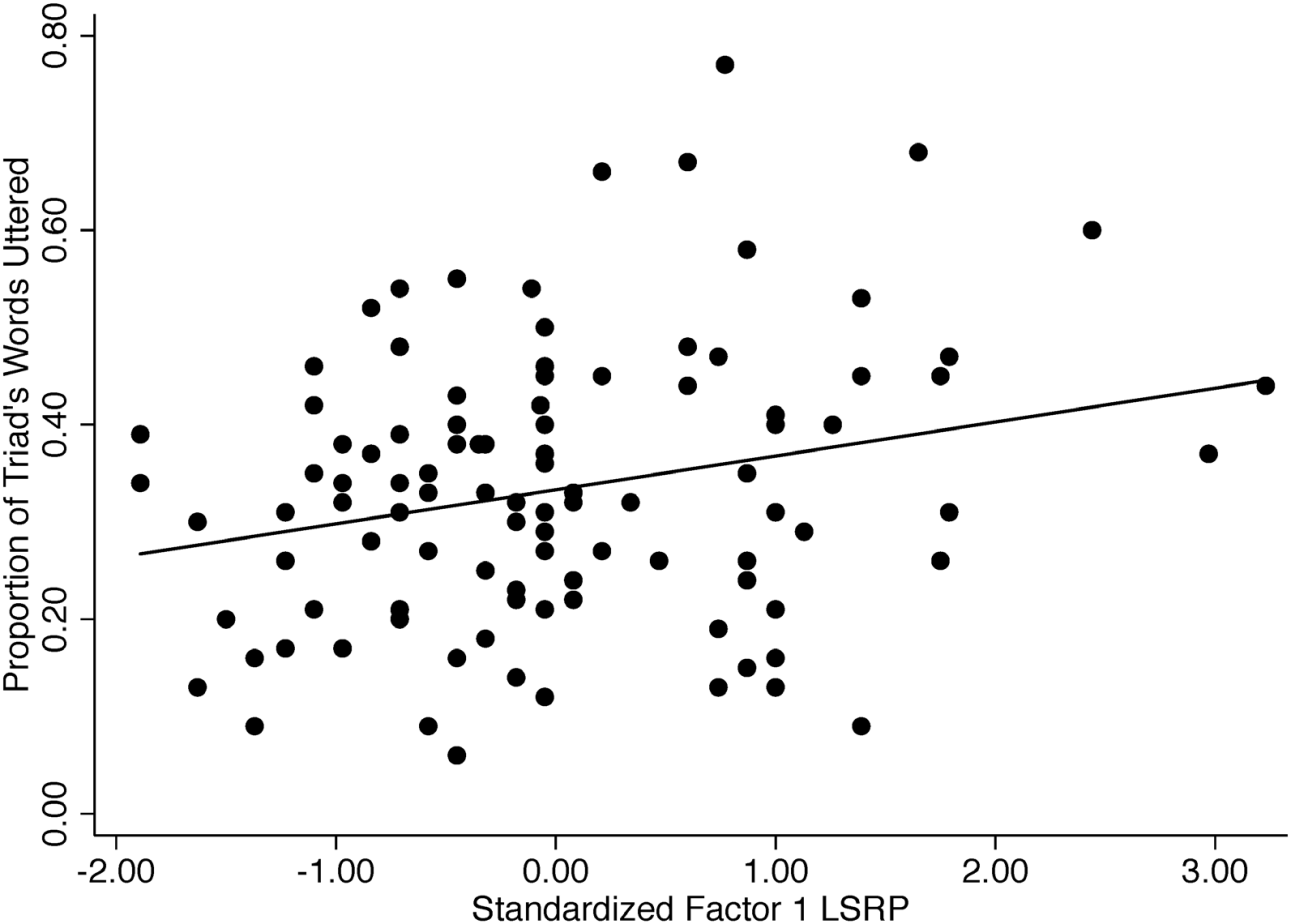
Regression of proportion of words uttered on F1 LSRP (psychopathy) score. *N* = 105.

Individuals with higher F1 LSRP scores produced a higher proportion of their conversational triad’s words (*N* = 105, β = 0.035±0.012, *P* = 0.005; Figure 1) and a higher proportion of sequence starts (β = 0.050±0.021, *P* = 0.025), but did not produce more interruptions per minute (β = 0.155±0.10, *P* = 0.134), sequence starts per word uttered (β = 0.168±0.111, *P* = 0.144) or interruptions per word uttered (β = 0.087±0.116, *P* = 0.457). In contrast, when we carried out the same analyses using F2 LSRP scores, we found no relationship to any measure of conversational dominance (proportion of words: β = −0.011±0.012, *P* = 0.373; proportion of sequence starts: β = 0.003±0.018, *P* = 0.884; interruption rate: β = −0.006±0.055, *P* = 0.911). Moreover, a multiple regression analysis revealed that, with the other psychopathy factor held constant, the two factors had opposing significant effects on proportion of words produced (F1: partial β = 0.046±0.014, *P* = 0.002; F2: partial β = −0.029±0.013, *P* = 0.032). Primary psychopathy was positively associated, whereas secondary psychopathy was negatively associated, with proportion of words uttered. An analogous analyses of associations of the psychopathy factors with proportion of sequence starts produced a significant positive association with F1 LSRP, and a nonsignificant negative association with F2 LSRP (F1: partial β = 0.057±0.020, *P* = 0.008; F2: partial β = −0.019±0.012, *P* = 0.137).

The relationship between primary psychopathy and quantitative dominance was stronger than the relationship between class level and quantitative dominance (described above). A multiple regression of proportion of words uttered on relative class level (as a dichotomous variable: most senior or not) and F1 LSRP score revealed a significant effect of Factor 1 LSRP score with relative class level held constant (*N* = 69, β = 0.038±0.01, *P* = 0.002), but no effect of relative class level controlling for F1 LSRP (β = 0.064±0.04, *P* = 0.130).

When we regressed the aggregate (i.e., by all three participants) number of words produced per transcribed minute on the F1 LSRP score of the participant with the highest LSRP score in that triad, we found a trend toward a positive relationship (*N* = 35, β = 6.65±3.30, *P* = 0.074). No such relationship was found with F2 LSRP (β = 1.74±5.92, *P* = 0.77).

In the pooled sample, there was no significant relationship between F1 LSRP score and use of affect words (*N* = 105, β = −0.26±0.22, *P* = 0.24); however, a model including (1) F1 LSRP, (2) sex and (3) their interaction as predictors revealed that (1) men used more affect words than women (β = −1.21±0.44, *P* = 0.007), (2) people higher on F1 LSRP used fewer affect words (β = −0.66±0.28, *P* = 0.02), and (3) the interaction term was not a significant predictor of affect word use (β = 0.67±0.45, *P* = 0.14). We found no relationships between F2 LSRP score and affect word use in either sex.

### Comparison among models using AIC

Among all possible bivariate and multivariate linear regression models, the lowest AIC value for predicting proportion of words produced included only two independent variables: F1 LSRP score (positive association) and F2 LSRP score (negative association). For predicting proportion of sequence starts per word uttered, two models yielded almost identical AIC values: a model including only childhood ZIP code median income (negative effect) and a model including F1 LSRP score (positive effect) and childhood ZIP code median income. For predicting interruptions performed per word uttered, no model approached statistical significance, so we did not compare AIC values among them.

## Discussion

Although a large literature has explored the manifestations of interpersonal dominance in face-to-face interaction [1], [14], little is known about the determinants of conversational dominance in non-task-centered zero-acquaintance situations. This is a noteworthy gap in knowledge, as many real-life social encounters with strangers occur in non-instrumental, apparently low-stakes contexts – yet these may in the aggregate have long-term socio-relational and psychological consequences. In addition, few studies have investigated the patterning of verbal aspects of naturalistic conversation from an evolutionary perspective. In this paper, we used recent theoretical and empirical work from evolutionary social psychology to formulate and test several hypotheses about the relationships between (1) physical, personality, and status-related characteristics and (2) aspects of conversational dominance.

Our study is the first to report an association between conversational dominance, or indeed any quantified measure of natural verbal behavior, and any measure of psychopathy. Specifically, we found that individuals higher in primary (F1) LSRP psychopathy produced a higher proportion of their conversation triad’s words, a higher proportion of sequence starts, and more interruptions per minute. We consider it highly unlikely that this association resulted from reverse causality (i.e., conversational dominance causing higher F1 LSRP scores) because (1) several studies have shown that the LSRP measures stable, real-world behavioral propensities (e.g. [33]) and (2) only three of the 16 F1 LSRP items are in any way related to verbal behavior. Furthermore, these findings are consistent with results linking psychopathy to a sense of grandiosity [27], self-perceived relative rank [37], and Social Dominance Orientation [39]. Elsewhere [36], we reported that, in a post-conversation cooperative dilemma, those higher in F1 psychopathy were more likely to defect on individuals who had interrupted them more during the conversation, suggesting that those higher on F1 prefer to form asymmetrical relationships in which they can dominate.

The function of conversational dominance for those higher in primary psychopathy remains an open question. Our findings are consistent with research linking F1 or similar psychopathy factors to verbal intelligence as measured by standardized tests [42], [43]. It is possible that individuals high in primary psychopathy are loquacious in any social situation, and this “glib charm” garners them social capital, which may be consistent with a strategy of pursuing status through prestige. However, in this same sample, we found that those higher in primary psychopathy were not more likely to receive cooperation from their conversation partners in an unannounced post-conversation social dilemma [36], possibly because interruptions were part of their conversational strategy. This suggests that others do not view such individuals as admirably successful models for deferential learning. One alternative is that individuals high in primary psychopathy specifically leverage opportunities to assess and manipulate new acquaintances by controlling conversations and gleaning useful information about them, that is, they use conversation as a means for exploitation, a pattern consistent with the pursuit of status through dominance.

Our experimental situation was framed as casual conversation, and thus lacked any overt cues of competition. Nonetheless, this situation affords proactive relational assessment. For example, conversational exchanges often included efforts by the speakers to identify common ground (e.g., shared academic majors, residence halls, etc.). We also found that F1 LSRP score was strongly negatively related to the probability of cooperating in the post-conversation prisoner’s dilemma, only when dyads discovered no common ground [36]. This suggests a readiness to defect on less valuable relationship partners (i.e., individuals less likely to be encountered again) – an exploitative orientation consistent with a preference for dominance. Two other effects are consistent with this strategic account: First, the aggregate word-production rate of triads was marginally greater as a function of the F1 LSRP score of the triad member with the highest F1 LSRP score. This suggests that conversations including people higher in primary psychopathy are generally “livelier” than conversations lacking such people. Thus, in non-directed zero-acquaintance small group situations such as our experiment (analogous to, for example, a real-world meet-and-greet context), individuals high in primary psychopathy may both produce and elicit a lot of “small talk”, and then subsequently use the information gathered for personal gain, disregarding the welfare of their interlocutors. Second, at least among males, those higher in primary psychopathy produced fewer affect words during the conversation, suggesting that their higher speech production was less about sharing their own appraisals than about extracting information from others.

Unlike primary psychopathy, secondary psychopathy did not correlate with conversational dominance in bivariate tests of association, and was negatively associated with proportion of words produced, holding constant the effects of F1 LSRP. This finding is consistent with the personality correlates of secondary psychopathy reported by Lynam and Derefinko [50]. We think that recently developed four-factor self-report psychopathy instruments (e.g. [62]) would reveal more finely differentiated relationships with conversational dominance in zero-acquaintance situations: specifically, we expect the interpersonal manipulation psychopathy factor to show the largest unique positive effect on conversational dominance.

One important direction for future research is to map the conditions under which primary psychopathic traits predict conversational dominance and the functional outcomes of floor and topic control. This should include studies across populations varying in the frequency of first acquaintance situations (e.g., relational mobility) and in conversational norms marking status asymmetries and power distance. Clinical psychopathy has been linked to insensitivity to social norms [28], predicting invariant behavior across contexts, but subclinical primary psychopathy can also be theoretically and empirically associated with increased sensitivity and strategic responsiveness to social constraints (see [36]). Further, little is known about inter-cultural differences in the expression of psychopathic traits (but see [63]), especially in small-scale societies, where notions of prestige and dominance, and even personality structure [64] may differ from those documented in other populations.

We found no evidence that physical formidability was associated with attainment of any form of conversational dominance in either sex (quantitative, participatory, or sequential). Physically formidable participants were not more likely to defect in the one-shot prisoner’s dilemma (PD) game that followed the conversation (unpublished data). Height and conversational dominance were not associated in either sex, despite potential taller individuals’ greater physical formidability (but see [65]) or hypothesized greater cognitive ability [66]. These null results are probably not attributable to a lack of statistical power, because we failed to find even suggestive trends toward associations between physical formidability and conversational dominance.

Two recently published sets of findings [26], [67] suggest that, even in social settings in which violence is culturally disvalued, male physical formidability affects sense of entitlement, competitiveness, and even views regarding egalitarianism and the justifiability of using military force. Our measurements of physical formidability were based solely on naïve respondents’ ratings of images of clothed bodies, as opposed to more direct assessments. However, we found moderately high inter-rater reliability for both our strength and fighting ability judgments. We also found a strong positive correlation between these two judgments, which were made by non-overlapping sets of respondents. Finally, Sell et al. [24] found that people are somewhat accurate (*r* = .66) at judging others’ physical strength based on images of bodies that did not include faces. (However, those stimulus images depicted shirtless men or women wearing standardized t-shirts – our stimulus images were of individuals wearing their own clothing).

Assuming that physical formidability does promote competitiveness and a sense of entitlement, our results indicate that our male conversation participants did not view their interaction as a competition for floor time or topic control. We suggest that both characteristics of our participant pool, and the experimental framing of the situation, account for the lack of relationships between male physical formidability and conversational dominance. Our participants, like those of Sell et al. [26] and Price et al. [67], were college students for whom status acquisition via physical intimidation is advantageous only under rare circumstances, which almost never include first-acquaintance situations governed by politeness norms [68]. In contrast to previous studies, our participants did not merely fill out questionnaires, but interacted face-to-face with peers. This interaction was specifically framed as “small talk among strangers”, and we provided generic instructions to discuss classes, majors, hometowns, sports, movies, “whatever”. This framing elicited a different set of motivations from the far more typical small group experiment, in which participants are asked to solve a specific problem or discuss a controversial topic (see references cited in [14]).

In our protocol, there was no exogenous incentive to compete for conversational control, nor to assert physical dominance in the pursuit of later advantage. Primary psychopathy, a personality trait associated with dominance striving, predicted conversational dominance, but physical traits associated with successful dominance attainment did not. This is understandable if individuals who are motivated to employ dominance-relevant strategies simultaneously recognized that (1) the experimental situation was governed by norms that sharply proscribe the use of physical intimidation, and (2) the experimental situation afforded the gathering of information that could subsequently be employed to exploit others. An important question for future research is thus the range of cultural contexts and situational frames that lead more formidable men to dominate conversations. Possibly, putatively egalitarian contexts in general reduce the effects of formidability on conversational dominance. This may occur both situationally and chronically across populations. Our participants were well-educated members of large-scale societies and therefore unrepresentative, in some respects, of humans generally [69]. Interactions among strangers in our study population may have been governed by norms of equality that have emerged only with the development of large-scale, complex, market-integrated societies [69].

We also found no relationship, even at the trend level, between facial attractiveness and any aspect of conversational dominance in either sex. This result was somewhat surprising, because (1) runaway intra-sexual prestige competition, based on physical attractiveness, is particularly prevalent in contemporary U.S. society [47], (2) female attractiveness differences are known to affect zero-acquaintance conversational behavior [70] and (3) our participants apparently did attend to each other’s attractiveness, because they were more likely to cooperate in the post-conversation PD toward more attractive co-participants, even after controlling for several other variables that affected PD play [36]. Again, we draw attention to the participant pool and the experimental framing: both may have foregrounded norms that prioritize intellectual ability over physical attractiveness.

With few exceptions, conversational dominance was also not predicted by other prestige markers (i.e., natal SES, visual cues of high status, or pursuing a demanding and lucrative academic major). Although we have treated physical height as a contributor to physical formidability, there is evidence that it also contributes to prestige-based social status (reviewed in [71]) however, as previously noted, height too did not predict conversational dominance, suggesting that, no matter the marker employed, in our experiment, conversational behavior was unaffected by prestige differences. Indeed, individuals from wealthier ZIP codes may have participated less than those from poorer ZIP codes, a result consistent with research [49] showing greater social disengagement in people of higher SES. One mechanism by which this could occur is contempt experienced by higher status individuals toward those perceived to be of lower status, and this could drive social disengagement – perceiving their lower-status compatriots as not meriting their attention, and having little of value to offer in the way of relationship opportunities, high-status individuals may simply remain disdainfully quiet. We did not measure either contempt toward co-participants, or self-evaluated social status, so we are unable to test this hypothesis.

We found only one tentative result suggesting positive relationships between prestige markers and conversational dominance. More senior students uttered more words than more junior students. This finding is consistent with our interpretation that the “small talk” frame affected the interaction. Our participants’ conversations mostly focused on navigating the university’s academic and social landscape, and, in this domain, the formers’ greater store of knowledge may have led their co-participants to grant them greater prestige. This interpretation raises the possibility that a complete account of the determinants of conversational dominance will need to partition its sources into those based on individual-level dominance (i.e., taken by dominant individuals) and those based on prestige (i.e., conferred by subordinates).

## Supporting Information

Text S1 **Rater samples, rater stimuli, and rating procedures.**
(PDF)

Text S2 **Analyses of upper body clothing type and formidability ratings.**
(PDF)
