# Supplementary material for: Subclinical Primary Psychopathy, but Not Physical Formidability or Attractiveness, Predicts Conversational Dominance in a Zero-Acquaintance Situation
Source: PLoS One. 2014 Nov 26;9(11):e113135. doi: 10.1371/journal.pone.0113135 (PMC4245099; doi:10.1371/journal.pone.0113135)
Supplement: Text S1 — Rater samples, rater stimuli, and rating procedures. (PDF) [file pone.0113135.s001.pdf]

Supplementary Text S1. Rater Samples, Rater Stimuli, and Rating Procedures

Characteristics of rater samples

The attractiveness raters (N = 70, 70% female) were recruited from the same introductory course participant pool as the conversation participants, during the academic year following the completion of the conversation trials. In addition to course credit, attractiveness raters received a $6 payment. Attractiveness raters were not asked their age or ethnicity. They were asked to decline to rate any individuals that they knew. Participants declined to rate facial photos in 8/1452 (0.55%) cases.

The formidability raters (N = 353, 56.9% female, mean age = 32.3 years, SD = 11.8) were recruited by posting notices on craigslist.org in 43 U.S. cities over a 29-day period in Sep-Oct 2010. Respondents were directed to a SurveyMonkey survey, at the end of which they were given the option to enter their email addresses in a raffle to win a $100 Amazon.com gift certificate.

The prestige raters (N = 8, gender data not collected) were recruited by four undergraduate research assistants from among their acquaintances. The social status raters (N = 35, 80% female) were recruited from the same introductory course participant pool as the conversation participants, during the second

academic year following the completion of the conversation trials. In addition to course credit, social status raters were offered a $3 payment.

Preparation of Stimuli for Raters, and Rating Procedures

Stimuli presented to the attractiveness raters were facial images prepared by cropping to an oval shape that included the participant's face and ears with as little hair as possible. Attractiveness ratings were collected using SuperLab 4.0. Raters were asked to judge each of 21 facial photographs for attractiveness on a 6-point Likert scale.

Stimuli presented to the formidability raters were prepared full-body images (1) cropped in the horizontal direction to minimize the presence of background cues to height, (2) cropped at the lower margin up to the border of the participant's feet and (3) de-identified by blurring the face in order to protect participants' privacy during online presentation. Sell et al. [1] found that people are significantly accurate at judging physical strength from images of bodies lacking identifiable faces.

Formidability raters were asked their gender and birth month (the latter question served as a random assignment device). Based on their answers, each rater viewed images of one-third (20 if female, 15 if male) of the conversation participants of their own gender. Each rater had a 0.50 probability (based on birth month) of being

asked to judge each pictured person's physical strength compared to that of other people of the same age and sex, and a 0.50 probability of being asked to judge the likelihood that each pictured person would win a physical fight against another person of the same age and sex. Participants made judgments on a 7-point Likert scale. To control for order effects, we used three different stimulus orders for each of the four rater groups (male-physical strength, female-physical strength, male-fighting ability, female-fighting ability). We substituted a new stimulus order after roughly one-third, and then again after roughly two-thirds, of the raters of each group had completed their ratings.

Each prestige rater was supplied with a set of randomly ordered cards, on each of which was printed the name of one of the 39 academic majors (including 7 double majors and the category "Undeclared") that at least one conversation participant had stated was his or her major. Of the 105 conversation participants, 99 (94%) announced their major during the conversation. The prestige raters were instructed to sort the majors in order of prestige and to write, on another card, their own definition or understanding of the concept "prestige." Prestige raters returned their sorted cards anonymously.

Each social status rater was shown 21 pairs of modified images of conversation co-participants (one pair from each of 21 conversation triads). The images were the same as those used for the physical formidability ratings, i.e., with the face blurred

to be unrecognizable. The prompt read “Please compare these two people in terms of *social status* (position in society)” along with a 7-point Likert scale. The left-right ordering of images was counterbalanced across the 6-7 presentations of each pair of images, and the presentation order of images was randomized for each rater.

We also obtained height estimates for each conversation participant. The wall against which they had stood to be photographed was marked with metric measurements and photographed. A research assistant compared the photograph of each participant with the photograph of the marked wall and produced a height estimate. Author MG, working independently, estimated the height of 42 of the 105 conversation participants.

References

1. Sell A, Cosmides L, Tooby J, Sznycer D, von Reuden C, et al. (2009) Human adaptations for the visual assessment of strength and fighting ability from the body and face. Proceedings of the Royal Society of London B 276: 575-584. doi:10.1098/rspb.2008.1177
