# Supplementary material for: Subclinical Primary Psychopathy, but Not Physical Formidability or Attractiveness, Predicts Conversational Dominance in a Zero-Acquaintance Situation
Source: PLoS One. 2014 Nov 26;9(11):e113135. doi: 10.1371/journal.pone.0113135 (PMC4245099; doi:10.1371/journal.pone.0113135)
Supplement: Text S2 — Analyses of upper body clothing type and formidability ratings. (PDF) [file pone.0113135.s002.pdf]

Supplementary Text S2. Analyses of Upper Body Clothing Type and Formidability Ratings

Rationale

In Sell et al's [1] tests of observers' abilities to judge physical formidability based on appearance, male targets were photographed while shirtless, while female targets were photographed wearing standardized white t-shirts. Because our participants wore their own clothes, uncontrolled variation in clothing may have affected (1) raters' assessments of physical formidability, and therefore (2) apparent statistical relationships between formidability and conversational dominance. In view of ancestral human fighting tactics, upper-body strength is the principal determinant of physical formidability [2]. Therefore, the most likely source of noise in our formidability measurements is variation in the amount of exposed upper-body skin. Specifically, it is plausible that when raters viewed images of participants displaying less upper-body skin (and therefore fewer cues to strength or fighting ability), they were more likely to rate them as intermediate, thereby artifactually reducing variance in preceived formidability. Here, we examine our formidability data for differences in both variance and mean rating values as a function of upper-body clothing type (tank-top, short-sleeved, and long-sleeved). We also re-calculate the relationships between formidability and conversational dominance using restricted samples of conversation participants, for which upper-body clothing type was invariant.

Results

Men

No men wore tank-tops. Men wearing long-sleeved shirts (N = 10) showed marginally less variation in perceived strength than men wearing long-sleeved shirts (N = 35) (SD of short-sleeved = 1.09, SD of long-sleeved = 0.62, variance ratio test, one-tailed P = 0.04). No difference in variation was found with respect to perceived fighting ability (SD of short-sleeved = 1.07, SD of long-sleeved = 0.74, one-tailed P = 0.12). Short-sleeved and long-sleeved men did not differ in mean perceived strength (t-test with unequal variances, $M \pm SE_{short}$ = 0.05 ± 0.18, $M \pm SE_{long}$ = -0.16±0.20, two-tailed P = 0.43) or mean perceived fighting ability (t-test with equal variances, $M \pm SE_{short}$ = 0.07 ± 0.18, $M \pm SE_{long}$ = -0.25±0.23, two-tailed P = 0.38).

These results show that among males, our measure of perceived strength (though not perceived fighting ability) was affected by clothing type. Table 1 shows relationships between perceived strength and conversational dominance for all males (unshaded rows; reproduced from Table 1 of the main article) and relationships between perceived strength and conversational dominance for only the 35 short-sleeved males (shaded rows).

Sample	Dependent variable	β ± robust SE	95% CI
All males	Words[a]	0.02 ± 0.02	-0.03-0.07
Short-sleeved males	Words[a]	0.01 ± 0.03	-0.04-0.06
All males	Interruptions[b]	0.17 ± 0.12	-0.08-0.41
Short-sleeved males	Interruptions[b]	0.20 ± 0.15	-0.12-0.53
All males	Sequence starts[c]	0.04 ± 0.03	-0.02-0.10
Short-sleeved males	Sequence starts[c]	0.03 ± 0.03	-0.03-0.08
All males	Interruptions/words[d]	0.07 ± 0.16	-0.25-0.40
Short-sleeved males	Interruptions/words[d]	0.14 ± 0.22	-0.33-0.61
All males	Sequence starts/words[e]	0.12 ± 0.12	-0.15-0.38
Short-sleeved males	Sequence starts/words[e]	0.07 ± 0.15	-0.24-0.39

Table 1. Linear regressions of conversational dominance variables on male perceived strength (standardized). For All males, N = 45 individuals. For Short-sleeved males, N = 35.

[a]proportion of triad's words uttered

[b]interruptions per transcribed minute, Box-Cox transformed

[c]proportion of triad's sequence starts

[d]interruptions performed per word uttered × 100, Box-Cox transformed

[e]sequence starts per word uttered × 100

<u>Women</u>

Among the 60 female participants, 9 wore tank-tops, 30 wore short-sleeved tops, and 21 wore long-sleeved tops. There were no significant differences among these three groups with respect to variance in perceived strength or fighting ability. However, tank-top wearers were perceived as lower in strength (t-test with equal variances, $M \pm SE_{tank\text{-}top}$ = -.66 ± 0.31, $M \pm SE_{short}$ = -0.02±0.16, two-tailed P = 0.06) and fighting ability ($M \pm SE_{tank\text{-}top}$ = -1.00 ± 0.27, $M \pm SE_{short}$ = 0.21±0.16, two-tailed P = 0.005) compared to short-sleeved women, and tank-top wearers were also perceived as lower in strength ($M \pm SE_{tank\text{-}top}$ = -.66 ± 0.31, $M \pm SE_{long}$ = 0.32±0.24, two-

tailed $P = 0.03$) and fighting ability ($M \pm SE_{tank\text{-}top} = -1.00 \pm 0.27$, $M \pm SE_{long} = 0.12 \pm 0.23$, two-tailed $P = 0.008$) compared to long-sleeved women. Short-sleeved and long-sleeved women did not differ in perceived strength ($M \pm SE_{short} = -0.02 \pm 0.16$, $M \pm SE_{long} = 0.32 \pm 0.24$, two-tailed $P = 0.23$) or perceived fighting ability ($M \pm SE_{short} = 0.21 \pm 0.16$, $M \pm SE_{long} = 0.12 \pm 0.23$, two-tailed $P = 0.71$).

Excluding the 9 tank-top clad women from analyses of the relationships between formidability variables and conversational dominance variables had little effect on the results. Only one association because marginally statistically significant, the one between perceived strength and proportion of sequence starts ($N = 51$, $\beta = 0.049 \pm 0.025$, $P = 0.052$).

References

1. Sell A, Cosmides L, Tooby J, Sznycer D, von Reuden C, et al. (2009) Human adaptations for the visual assessment of strength and fighting ability from the body and face. Proceedings of the Royal Society of London B 276: 575-584. doi:10.1098/rspb.2008.1177

2. Sell A, Hone LSE, Pound N (2012) The importance of physical strength to human males. Human Nature 23: 30-44.
